# The Utility of Pain Scale to Assess Verifiable vs Non-Verifiable Pain in United States Emergency Departments

**DOI:** 10.5811/westjem.2020.11.49030

**Published:** 2021-01-29

**Authors:** K. Tom Xu, James E. Morris, Christopher Piel

**Affiliations:** Texas Tech University Health Sciences Center, Department of Surgery, Division of Emergency Medicine, School of Medicine, Lubbock, Texas

## Abstract

**Introduction:**

We sought to examine the utility of self-reported pain scale by comparing emergency department (ED) triage pain scores of self-reported but non-verifiable painful conditions with those of verifiable painful conditions using a large, nationally representative sample.

**Methods:**

We analyzed the National Hospital Ambulatory Medical Care Survey (NHAMCS) 2015. Verifiable painful conditions were identified based on the final diagnoses in the five included International Classification of Diseases 9th revision codes. Non-verifiable painful conditions were identified by the five main reasons for visit. Only adults 18 years of age or older were included. The primary outcome variable was the pain scale from 0 to 10 at triage. We performed descriptive and multivariate analyses to investigate the relationships between the pain scale and whether the painful condition was verifiable, controlling for patient characteristics.

**Results:**

There were 55 million pain-related adult ED visits in 2015. The average pain scale was 6.49. For verifiable painful diagnoses, which were about 24% of the total visits, the average was 6.27, statistically significantly lower than that for non-verifiable painful conditions, 6.56. Even after controlling for the confounding of patient characteristics and comorbidities, verifiable painful diagnoses still presented less pain than those with non-verifiable painful complaints. Older age, female gender, and urban residents had significantly higher pain scores than their respective counterparts, controlling for other confounding factors. Psychiatric disorders were independently associated with higher pain scores by about a half point.

**Conclusion:**

Self-reported pain scales obtained at ED triage likely have a larger psychological component than a physiological one. Close attention to clinical appropriateness and overall patient comfort are more likely to lead to better health outcomes and patient experiences than focusing on self-reported pain alone.

## INTRODUCTION

It is well established that pain is both physiological and psychological.^1^–^4^ Treating pain has been aggressively emphasized by hospitals and particularly emergency departments (ED) since the late 1980s, and self-reported pain scales have been treated as the fifth vital signs.^5^,^6^ Since the early 1990s, the Joint Commission on Accreditation of Healthcare Organizations (JCAHO) and the Veterans Health Administration have promoted adequate pain control as a quality measure.^7^ In 2005, the American Pain Society published guidelines recommending that pain needed to be assessed and promptly treated in various settings.^8^

Because of these efforts, nearly all EDs in the US use some variations of self-reported pain scale at triage and likely again at nursing assessment. JCAHO recommended that extensive resources be used to assess and manage pain in the ambulatory setting.^9^ Many emergency physicians use the pain scale in their determination of diagnosis and decision to prescribe pain medications.^10^ The underlying but unverified assumption is that the reported pain scale has a clinical utility. In particular, if the pain scale were predictive of the severity of diseases and adverse clinical outcomes, the resources used to document and alleviate pain would be well justified. Unfortunately, few studies have addressed this issue. One study found that the reported pain scale did not predict patients’ desire for analgesia.^11^ Other studies showed that pain scale was not associated with vital signs in EDs^12^ or in prehospital settings.^13^

A recent study demonstrated that a high initial pain score did not predict the cause of pain identified by computed tomography, the need for admission, or surgery,^14^ suggesting that the severity of certain pathologies did not correlate well with reported pain. In contrast, in an earlier study of combat injuries, pain scale was significantly proportional to the severity of injuries, although not correlated with abnormal vital signs.^15^ This raises the question: If the pain scale correlates with the severity of disease in truly painful conditions, such as injuries, which can be verified by exam or imaging, how does it correlate with patients’ self-reported painful conditions with no verifiable painful diagnoses, such as non-specific abdominal or chest pain? Our objective in the current study was to compare the self-reported pain scales of verifiable painful conditions with those of non-verifiable painful conditions at ED triage among adults using nationally representative data of EDs in the US. Patient characteristics and comorbidities associated with the reported pain scales were also identified.

## METHODS

### Data

We analyzed data from the National Hospital Ambulatory Medical Care Survey (NHAMCS) 2015, representing about 137 million ED adult and pediatric visits (sample size n = 21,061) in the US in 2015. Key data elements of the NHAMCS included patient characteristics, visit characteristics, vital signs, tests and procedures performed, medications given, discharge status, up to five chief complaints and up to five diagnoses in *International Classification of Diseases 9^th^ revision* (ICD-9) codes. More details of the NHAMCS can be found at the US Centers for Disease Control and Prevention website (http://www.cdc.gov/nchs/ahcd.htm). Pain-related ED visits were extracted from the data and dichotomized into visits with verifiable and non-verifiable painful conditions.

Verifiable painful conditions were identified based on the final diagnoses in the five included ICD-9 codes: injuries to various body parts; acute myocardial infarction (AMI); nephrolithiasis/ureterolithiasis; and intestinal obstruction.^10^,^16^ Due to the small cell sizes for isolated intrathoracic, abdominal and pelvic injuries, respectively, they were grouped into one category. We created an additional category of multisystem injuries to encompass injuries that involved more than one body part.

The NHAMCS extracted the free text of the five main reasons for a visit and standardized the text into codes. Symptoms, including pain and injuries, accounted for over 90% of all ED visits.^17^ Following prior studies using the NHAMCS data to study pain-related visits,^16^,^18^ pain-related descriptors in the main reasons for a visit included pain, tenderness, burning or stinging, soreness, ache, cramps, spasms, discomfort, and injuries. For visits with self-reported pain from multiple body parts in chief complaints, an additional category was created. If any of the five main reasons reported was an injury, the visit was classified as injury-related, regardless of the remaining main reasons for the visit. A visit was considered having verifiable painful conditions if it had the previously described painful diagnoses, regardless of whether the main reasons for the visit were pain-related or not.

**Population Health Research Capsule**

What do we already know about this issue?

*Nearly all emergency departments (ED) use subjective pain scales at triage. Several studies have showed that the clinical relevance of pain scales is limited*.

What was the research question?

How do self-reported pain scales of non-verifiable painful conditions compare with those of verifiable painful conditions in adult ED visits?

What was the major finding of the study?

*The self-reported pain scale was higher for non-verifiable painful conditions than that for verifiable painful diagnoses*.

How does this improve population health?

*Understanding the limited utility of pain scales helps to more efficiently allocate resources for managing pain, which has been recognized as a public health challenge*.

### Inclusion and Exclusion Criteria

We included an ED visit in the sample for analyses if one of these criteria was met: 1) one of the five self-reported main reasons for the visit was related to pain or injuries; 2) the pain scale (0–10) at triage was >0 regardless of the main reasons for the visit; and 3) one or more of the final diagnoses was a verifiable painful condition as previously defined. The following visits were excluded: 1) persons younger than 18 years of age; 2) pain scale was not reported; and 3) arrival by ambulance/emergency medical services (EMS) because whether pain medications were given by EMS was not included in the data.

### Statistical Analyses

The dependent variable was the pain scale from 0 to 10 at triage. A patient’s age, gender, race/ethnicity, metropolitan statistical area (MSA) status, and geographic region of the ED were also included to examine the independent effect of verifiable vs non-verifiable painful conditions. The inclusion of these variables was based on findings from the studies cited previously.^3^,^10^,^12^,^13^ Chronic diseases have been demonstrated to be associated with pain.^19^–^21^ Several chronic comorbidities were included in the analyses: diabetes; asthma; chronic obstructive pulmonary disease, coronary artery disease, depression, hyperlipidemia, hypertension, and substance abuse. The NHAMCS included a total of 22 chronic conditions. Chronic comorbidities with a sample proportion <5% were not included in the multivariate analyses as independent variables.

We first calculated the average pain scales by verifiable and non-verifiable conditions, respectively. Descriptive statistics were obtained for patient characteristics and comorbidities. Multivariate regressions were performed to control for the possible confounding of patient characteristics and comorbidities. We used two models. The first model used a single dummy variable to represent all verifiable painful diagnoses and contrasted it with all non-verifiable conditions. To provide more details in regard to which specific conditions were different, a second model used non-verifiable abdominal pain, the most common reason for ED visits, as the comparison group, and contrasted each individual non-verifiable and verifiable conditions again this group. To further examine the consistency of the estimates obtained by the two models we used two specifications under each model: one included the comorbidities, while the other did not. To provide nationally representative estimates, the complex sampling design of the NHAMCS was controlled for in all analyses. We used statistical software Stata (StataCorp, College Station, TX). Statistical significance was defined as *p* < 0.05.

## RESULTS

**Figure 1:**
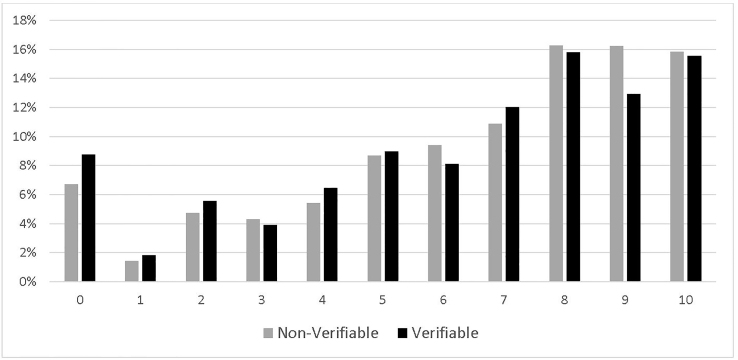
Pain scale: non-verifiable versus verifiable conditions.

There were a total of 105 million adult ED visits in the US in 2015, among which about 55 million were related to painful conditions. The average pain scale was 6.49. For verifiable painful diagnoses, which were about 24% of the total visits, the average was 6.27, statistically significantly (*p* < 0.05) lower than that for non-verifiable painful conditions, 6.56. Figure 1 illustrates the distributions of the pain scale for both groups. Noticeably, there were higher proportions of pain scales of 8, 9 and 10 for non-verifiable painful conditions than those for verifiable painful diagnoses.

**Figure 2:**
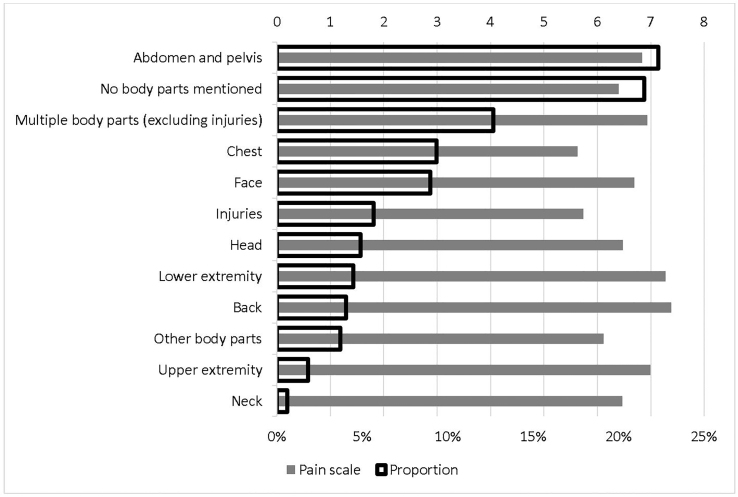
Painful chief complaints: proportion and pain scale.

**Figure 3:**
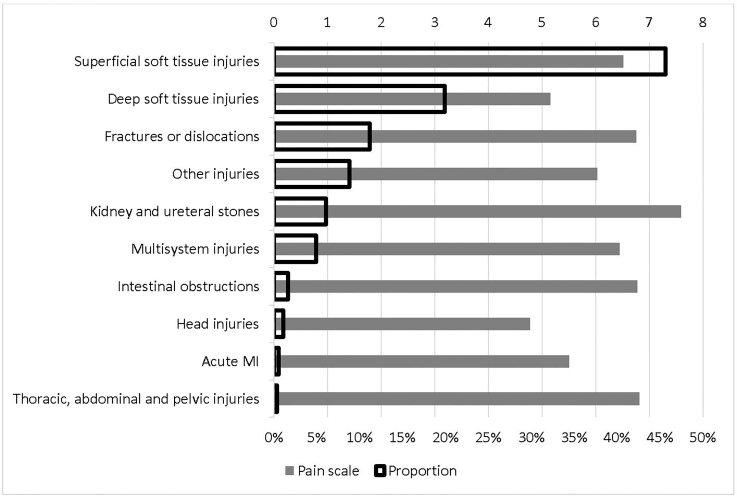
Verifiable painful diagnoses: proportion and pain scale.

Figure 2 reports the detailed average pain scale for each non-verifiable painful condition and the corresponding proportion. Back pain that was not related to injuries had the highest pain scale, 7.38, followed by leg pain that was not related to injuries, 7.27. The lowest pain scale was for chest pain that was not related to injuries, 5.63, followed by injuries, 5.74. As expected, pain in abdomen and pelvis had the highest proportion in main reasons for visits, 22.31%. Figure 3 is the counterpart of Figure 2 for verifiable painful diagnoses. The most painful diagnosis was kidney and ureteral stones, with an average pain scale of 7.59, followed by injuries to thorax, abdomen, or pelvis, 6.81. The lowest was head injuries, 4.77, followed by deep soft tissue injuries, 5.16. The highest proportion of verifiable painful diagnoses was superficial soft tissue injuries, 45.62%, followed by deep soft tissue injuries, 19.92%.

Descriptive statistics of patient characteristics and comorbidities are shown in Table 1. About 13.70% were elderly patients and 58.77% were females. Slightly less than two-thirds were non-Hispanic Whites, and non-Hispanic Blacks were about 21.04%. The majority, 84.03%, were visits made in urban EDs. About 35.70% visits were in the South region and 16.98% in the Northeast. Over one-fourth of patients had hypertension, 12.12% had diabetes, and 10.17% had depression.

Table 2 reports the results from multivariate regressions. Of note, the estimates are very consistent in both the direction (positive or negative association) and the magnitude, regardless of the models and specifications. Controlling for the confounding of patient characteristics, on average, verifiable painful diagnoses presented with 0.185 (*p* = 0.04) less pain on the 0–10 scale at ED triage. Compared to non-verifiable abdominal pain, non-verifiable chest pain, pain from injuries, pain with no body part mentioned reported significantly lower pain scores, whereas non-verifiable back and leg pain reported higher pain scores. Among verifiable painful diagnoses, deep soft tissue injuries, head injuries, and other injuries had significantly lower pain scores than non-verifiable abdominal pain, whereas kidney and ureteral stones had significantly higher pain scores.

Age, gender, and MSA were consistently significant and similar in magnitude across all model specifications. Controlling for other confounding factors, elderly persons reported a lower level of pain than their younger counterparts by −0.865 to −0.971, depending on the model and specification. Females had higher pain scores than males, by 0.233 to 0.331. Urban patients reported higher pain scores than rural patients, by 0.679 to 0.699. Interestingly, among comorbidities, psychiatric disorders, depression, and substance abuse, were independently associated with higher pain scores by 0.493 to 0.528, and 0.430 to 0.433, respectively. The only non-psychiatric comorbidity that was statistically significant was diabetes, with an average of 0.347 to 0.350 higher pain score than in non-diabetics.

## LIMITATIONS

There are several limitations of the current study. First, we used a nationally representative data set for secondary data analyses. All potential biases and pitfalls inherent to secondary data analyses are possible. Second, only adult ED visits were included to ensure the validity of the self-reported pain scale. Consequently, the results are not generalizable to the pediatric population. Third, we included only up to five diagnoses and five main reasons for visits in the data. More complex presentations and diagnoses may have been omitted but the proportion should be very small. Fourth, the exclusion of patients brought in by ambulance/EMS may have skewed the severity mix of the visits. As previously discussed, ambulance/EMS arrivals were excluded because whether pain medications were given en route was unknown. We compared the mean pain scales between the ambulance subsample with the sample included in the analyses, which were 6.05 and 6.49, respectively, and the *P* value for the difference was <0.01. This suggests that pain medications were likely given en route, as we would expect that EMS arrivals usually have higher acuity and severity. In addition, we compared the mean pain scores between verifiable conditions (mean = 5.88) and non-verifiable conditions (mean = 6.14) within the ambulance subsample. The difference was not significant, possibly due to a much smaller sample size of ambulance arrivals.

## DISCUSSION

This is the first study that compared self-reported pain scales at ED triage between verifiable painful diagnoses and non-verifiable painful chief complaints in adult ED visits using a large, nationally representative data set. It is interesting to note that patients with non-specific pains, such as non-traumatic abdominal, back and leg pain, had higher self-reported pain scores than those with fractures and bowel obstruction. This points to the possibility that in the ED setting, self-reported pain scale may have a much larger psychological component than previously thought. The large psychological component is further illustrated by the independent effects of depression and substance abuse on elevated self-reported pain scale found in this study, confirming the results from prior research indicating that substance abusers and patients with psychiatric comorbidities experience higher levels of pain.^22^–^24^ Patients with a chronic pain diagnosis usually have psychological diagnoses, and among them, ED patients were found to have a higher propensity for opioid abuse than pain clinic patients.^25^,^26^ In addition, personality disorders, anxiety, and panic attack were diagnoses more commonly associated with aberrant prescription behaviors.^26^

Understanding this association is particularly important in deciding how to treat non-verifiable painful complaints as the medical community is turning away from opioid-based treatments for pain. The key findings from the current study provided another piece of evidence showing that opioids may not be effective in treating non-verifiable painful conditions because of its large psychological component. Alternative and non-addictive treatment options need to be explored. Research has demonstrated the safety and efficacy of non-opioid therapies, including ketamine,^27^ metoclopramide for acute migraine headache,^28^ and other targeted therapies such as ketorolac for renal colic.^29^ These therapies can lead to a significant reduction in opioid use^30^ without leading to decreases in patient satisfaction.^31^

Physicians have been under increased scrutiny to provide adequate analgesia to patients for the past 20 years.^32^ There have even been initiatives to match opioid analgesia to specific pain intensities,^33^ despite findings showing that demographic factors such as race, age, insurance, and ED utilization lend to variability in self-reported pain scoring.^34^ Furthermore, pain scores do not accurately reflect ED patient experience or correlate well with the appropriateness of triage and treatment decisions.^35^ In fact, one study found that patient-reported visual analog pain scales were not indicative of their desire for analgesia among those with acute pain.^11^ These factors have important implications in physician’s decision-making regarding pain management in the ED. If self-reported pain does not correlate with the severity of disease or health outcome,^14^ strategies for more efficient use of resources need to be developed. More focus should be put on the overall patient comfort with less emphasis on pain scores.

## CONCLUSION

The current study used a large, nationally representative ED sample to demonstrate the limitation of self-reported pain scores in the ED setting. In particular, pain scales obtained at triage likely have a larger psychological component than a physiological one, as the self-reported pain score is higher in non-verifiable painful conditions than that in verifiable painful conditions. Close attention to clinical appropriateness and overall patient comfort are more likely to lead to better health outcomes and patient experiences than focusing on self-reported pain alone.

## Figures and Tables

**Table 1:** Patient characteristics.

Patient characteristics	Proportion %
Age ≥ 65 years	13.70
Female	58.77
Race/ethnicity	
Non-Hispanic white	61.21
Non-Hispanic black	21.04
Hispanic	15.24
Other races	2.50
MSA	84.03
Region	
Northeast	16.98
Midwest	24.84
South	35.70
West	22.49
Comorbidities	
Diabetes	12.12
Asthma	10.13
COPD	5.05
CAD	5.97
Depression	10.17
Hyperlipidemia	9.15
Hypertension	27.07
Substance abuse	7.48

*MSA*, metropolitan statistical area; *COPD*, chronic obstructive pulmonary disease; *CAD*, coronary artery disease.

**Table 2:** Results from multivariate regressions.

	Model 1				Model 2			
	
	Est.	p	Est.	p	Est.	p	Est.	p
Verifiable Dx	−0.185	0.04	−0.155	0.08				
Pain in CC (vs. abdominal pain in CC)								
Pain of head					−0.272	0.25	−0.269	0.25
Pain of face					−0.153	0.42	−0.142	0.46
Pain of neck					−0.258	0.50	−0.215	0.58
Pain of chest					−1.049	0.00	−1.100	0.00
Pain of back					0.631	0.00	0.614	0.00
Pain of upper extremity					0.244	0.40	0.245	0.38
Pain of lower extremity					0.541	0.01	0.528	0.02
Pain of other body parts					−0.651	0.16	−0.683	0.15
Pain of multiple body parts (excluding injuries)					0.131	0.43	0.122	0.46
Pain from injuries					−0.956	0.00	−0.997	0.00
Pain but no body parts mentioned					−0.358	0.04	−0.417	0.02
Verifiable painful Dx (vs. abdominal pain in CC)								
Fractures or dislocations					0.191	0.43	0.208	0.38
Superficial soft tissue injuries					−0.221	0.17	−0.209	0.20
Deep soft tissue injuries					−1.476	0.00	−1.442	0.00
Head injuries					−1.828	0.02	−1.783	0.02
Thoracic, abdominal and pelvic injuries					0.109	0.94	0.199	0.88
Other injuries					−0.691	0.02	−0.686	0.02
Multisystem injuries					−0.143	0.73	−0.155	0.71
Acute MI					−0.794	0.31	−1.019	0.20
Kidney and ureteral stones					0.841	0.00	0.893	0.00
Intestinal obstructions					0.455	0.42	0.430	0.46
≥ 65 years old	−0.971	0.00	−0.877	0.00	−0.921	0.00	−0.865	0.00
Female	0.331	0.00	0.307	0.00	0.259	0.00	0.233	0.01
Race/ethnicity (vs. Non-Hispanic white)								
Non-Hispanic black	0.181	0.14	0.217	0.07	0.186	0.14	0.225	0.06
Hispanic	−0.188	0.19	−0.141	0.32	−0.205	0.15	−0.153	0.28
Other races	−0.450	0.16	−0.434	0.15	−0.438	0.15	−0.414	0.15
MSA	0.699	0.01	0.697	0.01	0.683	0.01	0.679	0.01
Region (vs Northeast)								
Midwest	−0.077	0.82	−0.092	0.78	−0.089	0.79	−0.113	0.73
South	0.181	0.55	0.196	0.51	0.171	0.57	0.185	0.53
West	0.011	0.98	0.009	0.98	0.017	0.96	0.018	0.96
Comorbidities								
Diabetes			0.347	0.02			0.350	0.02
Asthma			0.098	0.50			0.145	0.29
COPD			0.121	0.54			0.200	0.31
CAD			0.030	0.87			0.168	0.34
Depression			0.493	0.00			0.528	0.00
Hyperlipidemia			−0.303	0.10			−0.292	0.12
Hypertension			−0.142	0.19			−0.117	0.26
Substance abuse			0.433	0.01			0.430	0.01

*Dx*, diagnosis; *CC*, chief complaints; *MI*, myocardial infarction; *MSA*, metropolitan statistical area; *COPD*, chronic obstructive pulmonary disease; *CAD*, coronary artery disease.
